# Binary code similarity analysis based on naming function and common vector space

**DOI:** 10.1038/s41598-023-42769-9

**Published:** 2023-09-21

**Authors:** Bing Xia, Jianmin Pang, Xin Zhou, Zheng Shan, Junchao Wang, Feng Yue

**Affiliations:** 1 State Key Laboratory of Mathematical Engineering and Advanced Computing, Zhengzhou, China; 2 Zhongyuan University of Technology, Zhengzhou, China

**Keywords:** Computer science, Software

## Abstract

Binary code similarity analysis is widely used in the field of vulnerability search where source code may not be available to detect whether two binary functions are similar or not. Based on deep learning and natural processing techniques, several approaches have been proposed to perform cross-platform binary code similarity analysis using control flow graphs. However, existing schemes suffer from the shortcomings of large differences in instruction syntaxes across different target platforms, inability to align control flow graph nodes, and less introduction of high-level semantics of stability, which pose challenges for identifying similar computations between binary functions of different platforms generated from the same source code. We argue that extracting stable, platform-independent semantics can improve model accuracy, and a cross-platform binary function similarity comparison model *N_Match* is proposed. The model elevates different platform instructions to the same semantic space to shield their underlying platform instruction differences, uses graph embedding technology to learn the stability semantics of neighbors, extracts high-level knowledge of naming function to alleviate the differences brought about by cross-platform and cross-optimization levels, and combines the stable graph structure as well as the stable, platform-independent API knowledge of naming function to represent the final semantics of functions. The experimental results show that the model accuracy of *N_Match* outperforms the baseline model in terms of cross-platform, cross-optimization level, and industrial scenarios. In the vulnerability search experiment, *N_Match* significantly improves hit@N, the *mAP* exceeds the current graph embedding model by 66%. In addition, we also give several interesting observations from the experiments. The code and model are publicly available at https://www.github.com/CSecurityZhongYuan/Binary-Name_Match.

## Introduction

Binary code similarity analysis is used to determine whether binary functions compiled from the same source code are similar or different. Due to code reuse, the same source code can be compiled with cross-optimization levels -O[0, 1, 2, 3] to generate binary code of cross-platform such as X86 (a standardized numbering abbreviation for a family of intel general-purpose computers), ARM (Advanced RISC Machine, ARM), MIPS (Million Instructions Per Second, MIPS), the same binary code will appear in multiple released programs, or even in multiple parts of a program. Once a bug is found in a binary code, the similarity analysis techniques can be used to find the same bug code or similar bug code, so as to quickly and timely find the risk or vulnerability^1^. Since cross-platform code syntax varies greatly, such as *opcodes*, *operands*, *function calls*, and *memory accesses*, semantic similarity is usually computed on the comparison, and the best practice is to compare the similarity between the known binary code semantic and the unknown binary code semantic. David and Yahav^2^ calculates the number of identical strand code fragments in the two sets as the similarity. Huang et al.^3^ computes the similarity between the longest execution paths on the CFG (Control Flow Graph, CFG).

Recent research results [125] pointed out and verified that similarity results obtained by utilizing graph neural networks based on CFG perform best in binary code similarity comparison. The idea is that similar codes with less structural variation, structural stability, and semantic information from graphs, therefore, many scholars have utilized the stability of the CFG structure and the graph-carrying annotation semantics to represent binary code semantics. Xu et al.^4^ proposes a Gemini scheme based on manual feature extraction to compute binary function semantics with the help of graph neural networks by manually analyzing statistical and platform-related basic block features. The UFEBS (Unsupervised Features Extraction for Binary Similarity, UFEBS) scheme proposed in^5^ utilizes RNN (recurrent neural network, RNN) to compute the basic block semantics and converts CFGs into graph embeddings with the help of graph neural networks. Alrabaee et al.^6^ incorporate the properties of graphs to propose a semantically integrated graph containing CFG, call graph, and register flow graph. Qiu et al.^7^ incorporates inline functions and library functions called by CFG to propose Execution Dependence Graph. Zhang et al.^8^ use Reductive Instruction Dependent Graph.

### Problem definition

Given two binary functions from different platforms, our goal is to determine whether the two functions come from the same source code. Despite deep learning’s ability to capture rich code features and stable graph structure information, this problem still faces three technical challenges.

### Challenge 1: Instruction difference

The instruction difference of cross-platform varies greatly. Not only there is a huge syntactic gap between the instruction sets of binary generated by compiling the same source code for different platforms, but there are also differences in the real-value features extracted by manual means such as Gemini (“Model accuracy”). Different optimization options for the same platform can also cause instruction differences.

### Challenge 2: CFG difference

Taking the *EVP-PKEY-decrypt()* function in OpenSSL (Open Secure Sockets Layer, OpenSSL) as an example, which carries the *CVE-2021-3711* vulnerability. It is found that the function using IDA Pro analyzed carries 15 basic blocks on ARM platforms, 16 basic blocks on X86 platforms, and 13 basic blocks on MIPS platforms. Not only the differences in the number of basic blocks in the CFG, but the graph neural network that updates the states of nodes through neighboring nodes also affects the similarity comparison results, resulting in a higher FPR (False Positive Rate, FPR) when vulnerability matching (“Vulnerability search”).

### Challenge 3: Little knowledge coreference

Much high-level knowledge of functions that are stable is not considered. Current solutions generate function embedding from low-level instructions and graph structure perspective, without considering high-level semantics that do not change with the platform, such as *API (Application Programming Interface) names* carried by binary functions.

### Insights

It is well known that software is a new tool for the perpetuation and exchange of human knowledge. Similar to a human written language describing a work, software is created by humans with specific programming languages and knowledge. The knowledge contained in software is as limited as the knowledge described by natural language to reality constraints^9^ and practical exigencies, so software knowledge is stable, repeatable, and predictable^10^. For debugging and calling convenience, experienced software developers often give functions a *name* that represents their meaning, such as *SendData* may be on behalf of the function to *achieve sending data*. Software developers usually call an API or an implemented function within software code to speed up software iteration. These naming functions which are linguistically empowered by the developer’s human working experience on the software body, express the high-level semantics of the function. For binary code that contains a lot of low-level semantics such as *opcodes, registers, memory addresses*, these naming functions more clearly express the high-level semantics of a binary code.

**Figure 1:**
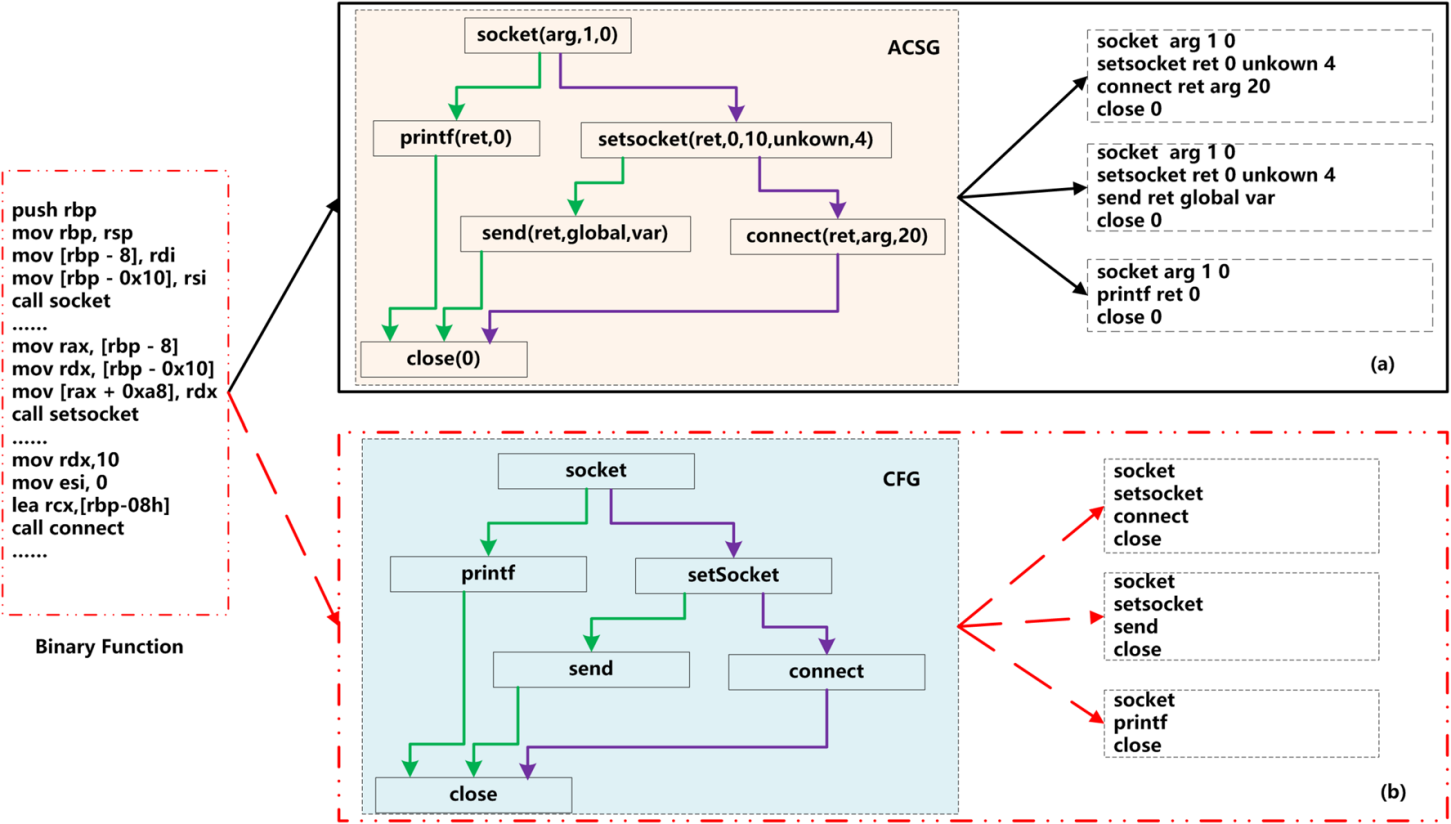
(**a**) ACSG using *callsite* sequence as naming function in^11^; (**b**) N_Match using *API* sequence as naming function.

Binary functions are usually stripped, and the debug information containing function names is removed entirely. To assign meaningful names for stripped binary code, David et al.^11^ proposed the concept of ACSG (Augmented Call Sites Graph, ACSG), each node ${callsite = (st_1,\ldots ,st_k,arg_1,\ldots ,arg_s)}$ in the ACSG is an API called by the binary code, where *st* is a subtoken sliced by API name, and *arg* is the parameter used by the API, as shown in Fig. 1a. According to^11^, the sequence of ${(callsite_1,\ldots ,callsite_n)}$ carried on binary code can represent names of binary functions. However, the ACSG has two shortcomings, one is inaccurate parameter recognition such as some parameter values are *unkonwn* (Fig. 1a), and the other is that it only supports the amd64 platform.

Inspired by the ACSG proposed by^11^, we refer to the API sequences that can be extracted from the binary code as *naming function* (Fig. 1b). Compared to the CFG, API is platform-independent and is not changed by the compiler or optimization, so the API sequence does not change with cross-platform, cross-compiler, and cross-optimization. Highly stable advanced knowledge apply to binary code similarity has several advantages. The more stable the naming functions, the smaller the distance between functions that come from the original code. The greater the variation in the naming functions, the greater the comparison distance between functions. At the same time, these naming function helps to explain clearly why they are similar, alleviating the current lack of deep learning interpretability. Lastly, compared to low-level assembly instructions, the naming function can deal with code obfuscation^11^.

### Our approach

Two binary functions from the same source code have small high-level semantic differences, despite the large differences in the syntax of the low-level instructions expressed across platforms. Based on this natural observation, we propose a new binary code similarity model N_Match, which shields the underlying instruction differences by the common vector space, learns the stability semantics of neighbors using graph embedding techniques, extracts *naming function* to shield the differences arising from cross-platform and cross-optimization levels, and is well suited to the three challenges mentioned above.

**Common vector space** for cross-platform instructions. Embedding refers to mapping an input space object *x* to another output space object *y*, making comparison result easy to compute. Since word embedding techniques automatically learn a distributed representation of a word form dataset, we trained a word embedding model *x2v* that maps instructions from different platforms to the same space representation, which shields the instruction differences of cross-platform and facilitates binary code similarity computation (“Common vector space embedding”) to address the challenge 1.

### Graph embedding

The structural information of graph stabilization helps to enhance the similarity. Based on the common vector space embedding, each instruction in the basic block is regarded as a word and the basic block as a sentence, and the basic block embedding is generated using the sequence representation learning with self-attention mechanism (“Basic block embedding”). Then basic block embedding and adjacency matrix of CFG are fed into the graph neural network, and finally, CFG generates a graph embedding (“Graph embedding”) to address the challenge 2.

### Naming function embedding

An API sequence of binary code is used as *naming function*. Each API is sliced to obtain *subtoken* sequence, and a subtoken is treated as a word, and the naming function is treated as a sentence, then using the sequence representation learning with a self-attention mechanism to generate naming function embedding (“Naming function embedding”). As the naming function does not change with the platform, it can shield the differences between the underlying different platforms to address the challenge 3 and significantly improve the accuracy of the code similarity metric (“Cross-platform comparison between the same optimization levels5.5”), and the experimental results show that the vulnerability query *mAP*(mean Average Precision) of N_Match is higher than the previous solutions such as Gemini^4^ and UFEBS^5^ (“Vulnerability search”).

### Main contribution

This paper makes the following contributions.

1. We propose a new binary code similarity model N_Match. The model combines graph embedding and naming function embedding, stable naming function semantics shields the differences of cross-platform and significantly improve the code similarity results.
2. We propose a naming function embedding method and build a pre-trained *k2v* dictionary. To adequately represent naming function embedding, we extract simple paths on the CFG and slice the API names carried on the paths as sentences. In this way not only can each subtoken learn the contextual semantics of the sequence, but also the sequence of subtokens on the simple path can reflect the dynamic properties of the function at a given execution moment, and can fully express the function of binary code, which significantly improved comparative accuracy.
3. We conduct several experimental tasks, and the results show that the model accuracy of N_Match outperformed the current model in terms of cross-platform, cross-optimization levels, and vulnerability search. The code and model are publicly available at https://www.github.com/CSecurityZhongYuan/Binary-Name_Match.

## Background

In this section, we provide the necessary background for N_Match, including word embedding models, graph neural networks, recurrent neural networks, and self-attention mechanisms.

### Word embeddings

Word2Vec is a neural networks language model proposed by Mikolov^12^, which also is a word embedding technique capable of converting a word into a vector representation. Word2Vec obtains word embedding $\vec {w}$ based on the context *context(w)* of the current word, so the model training goal is to maximize *p*($w_i$ $\mid$context($w_i$)). Word2vec has CBOW and Skip-gram word representation strategies, in which CBOW predicts the current word based on context, and Skip-gram predicts the context based on the current word. Since Word2Vec can map words to the common vector space and the distance between word vectors reveals the semantic relationship, the joint deep learning model can explore the potential relationship between sentences, which is widely used in the field of natural language processing.

### Graph neural networks

A binary function can be represented by a directed *CFG = (V, E)* equivalently, where *V* represents a set of vertices of basic blocks in the CFG, and edge *(u,v)*$\in$*E* denotes the *TRUE-FALSE* relationship between vertex *u* and vertex *v*. We do not distinguish such types of edges but treat all edges as simply a temporal sequential call relationship.

**Figure 2:**
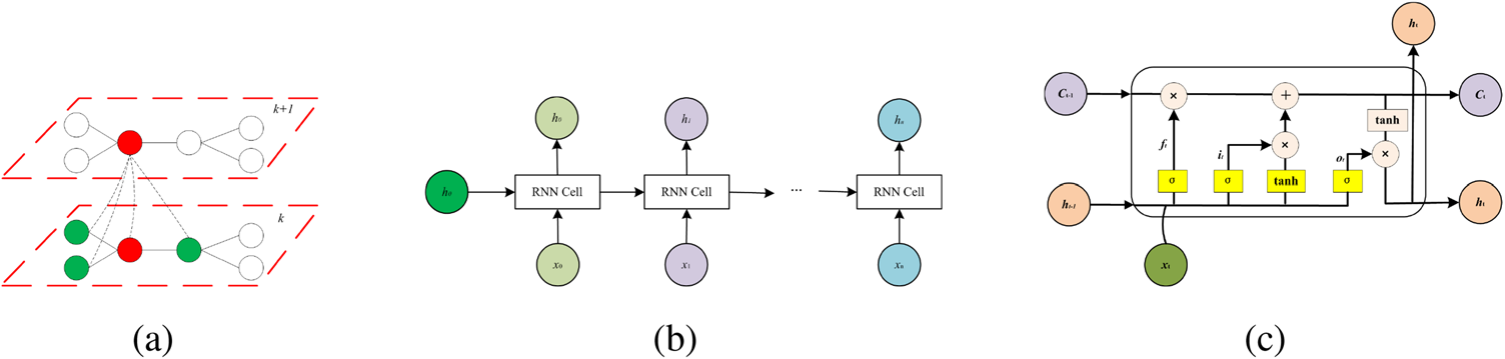
(**a**) Node updated by the neighbor node; (**b**) structure of RNN; (**c**) a cell of LSTM.

The graph neural network represented by Structure2Vec^13^ implements the update of the node *v* itself based on $N_{(v)}$, where $N_{(v)}$ representing the set of neighbors of node *v*. In each round of propagation operation, the network generates a new node representation based on the convergence of its own node features $\vec {h}_v$ and neighbors features $\vec {N_{(v)}}$. As shown in Fig. 2a, green nodes represent the neighbors of red nodes, and the red node feature value in the *(k+1)-th* round is a weighted average of red node feature value and green nodes feature values in the *k-th* round. Finally, after *k* rounds, all node features are aggregated as the final graph embedding result $G_{out}$.

### Recurrent neural networks

The RNN implements a sequence of input data to compute a fixed-length vector recursively. The input ($x_1,\ldots ,x_n$) represent a sentence, where $x_i$ denotes *i-th* word in the sentence. As RNN requires a fixed sentence length *n*, long sentences over *n* need to be truncated and sentences not exceeding *n* need to be padded. To generate the sentence learning representation, RNN needs to obtain each word embedding from the pre-trained word embedding dictionary $E^{d}_V$ via the *embedding* operation defined in (1), where *d* is word embedding size and *V* is the total number of words in the dictionary. Then a series of RNN^14^ units is used to encode the hidden states ($h_1,\ldots ,h_n$) .

1 $$\begin{aligned} {h_1,\ldots ,h_n} = RNN_{encoder}(embedding(E^{d}_V,x_i,\ldots ,x_n)) \end{aligned}$$

The basic structure of RNN is shown in Fig. 2b, which consists of an initial state $h_0$, an input time series $x_t$ and an output time series $h_t$. The final hidden state $h_n$ is a sequence embedding result. When a sentence is received, $h_n$ theoretically contains the meaning of a sentence. The LSTM^14^ (Long Short Term Memory, LSTM) model solves the problem of RNN short-term memory by adding a gates mechanism, allowing RNN to really make effective use of long-term information. An LSTM Cell at time *t* is shown in Fig. 2c, including the input data $x_t$ at time *t*, the cell state $C_t$, the hidden layer state $h_t$, the forgetting gate $f_t$, the memory gate $i_t$ and the output gate $o_t$. The implementation of each hidden state is to pass the remaining information obtained in the forgotten cell state, remember the new information, and compute the above information together with the current time input to generate the hidden layer state.

### Self-attention mechanisms

The attention mechanism was first proposed in [111], followed by [112] which applies the attention mechanism to the field of NLP. Our implementation uses a self-attentive mechanism proposed in^15^. Initializes randomly a vector as the query vector and performs a similarity calculation with each word in a sentence, then uses *softmax* to generate the attention weight. A weighted summation of the hidden states ($h_1,\ldots ,h_n$) is performed and the result is the output of the attention mechanism.

Neural networks can be well integrated with attention mechanisms that focus on the key information. During the basic block embedding process, the attention mechanism captures important semantic features of the basic block by scoring to focus attention on important instructions.

## Model overview

This section focuses on the overall framework of the N_Match model and the model evaluation criteria.

### Framework

For clarity of interpretation, we summarize and give all the notations used in this paper in Table 1. Meanwhile, the relationships between instruction, basic block, function respectively, and embedding learning are described in Table 1. Let us first describe the similarity problem for two cross-platform binary functions. Two binary functions $F_1$ and $F_2$ are similar^16^ meaning that the results $F^s_1$ and $F^s_2$ generated by compiling the same original source *s* are similar, noted as $F_1\simeq F_2$ . The compiler *c* (such as GCC, Clang) completes the conversion from source code *s* to binary function *F*. During the conversion process compiler *c* can be compiled to generate binary code for different platforms (e.g. X86, ARM, MIPS) and can carry different compilation levels -O[0,1,2,3]. In this way, a source code *s* can generate 12 binary functions on 3 platforms and 4 optimization levels.

**Table 1:** Notation definition.

Notation	Definition
*s*	Source code
*c*	Compiler
*F*	A binary function in object file
$\vec {F}$	Embedding vector of a function *F*
$F^s$	*F* compiled from source code *s*
*x2v*	Cross-platform instruction dictionary
*k2v*	Knowledge dictionary
*b*	Basic block
$\vec {b}$	Embedding vector of a basic block *b*
*i*	Instruction
$\vec {i}$	Embedding vector of an instruction *i*
$b^i$	Sequence of instructions in *b*
$x_i$	i-th instruction in $b^{i}$
$\vec x_{i}$	Embedding vector of $x_{i}$
*k*	A knowledge of naming function
$\vec {k}$	Embedding vector of knowledge *k*
$L^f$	Instruction length of *F*
$L^b$	Length of $b^{i}$
$F^k$	Sequence of knowledge in *F*
$L^k$	Length of knowledge in *F*
$\vec {F}^{k}$	Embedding vector of $F^{k}$
*g*	CFG of a function *F*
$L^{g}$	Number of basic blocks in *F*
$\vec {F}^{g}$	Embedding vector of *g*

**Figure 3:**
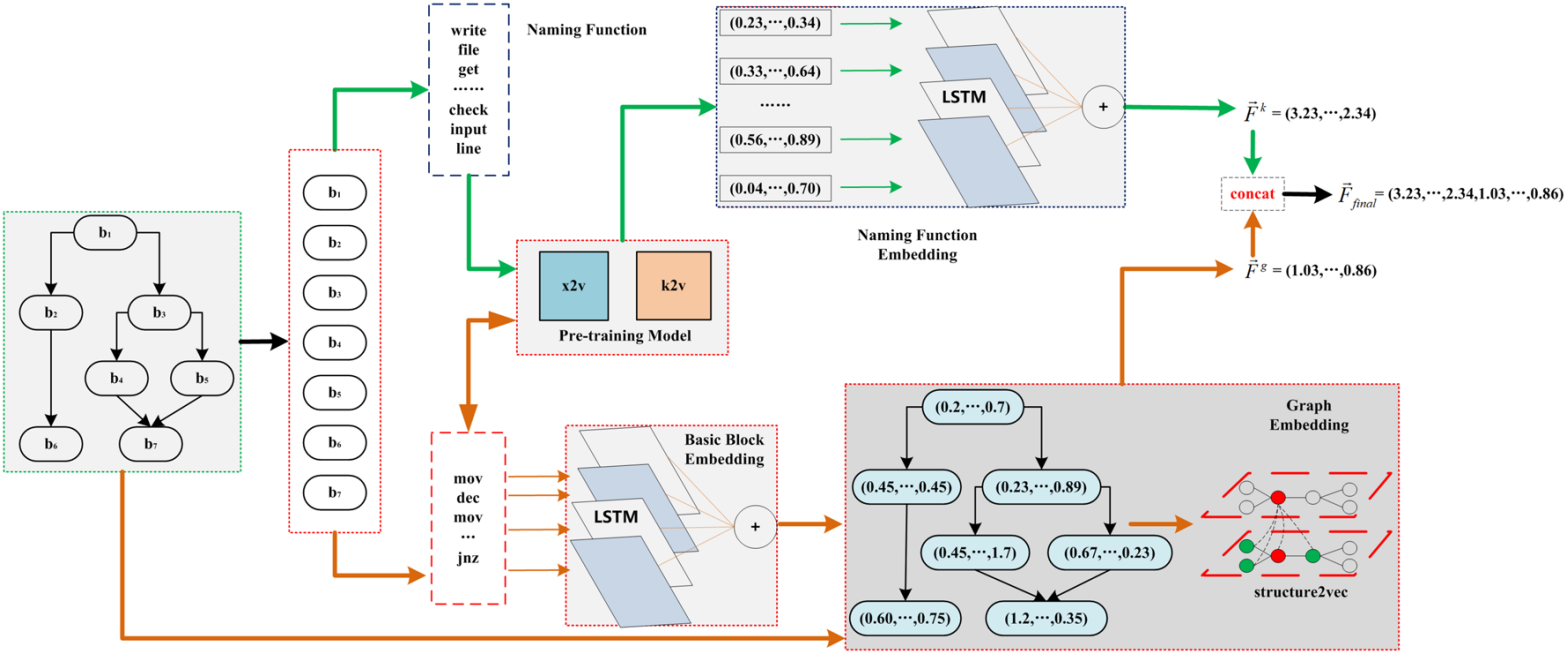
Architecture of N_Match.

We use the embedding model architecture to represent a binary function. As shown in Fig. 3, the overall framework of the model consists of naming function embedding and graph embedding. For a given binary function, naming function embedding online component and graph embeddings online component need to utilize pre-trained models offline component as dictionaries.

#### Graph embedding

To complete the process of converting a CFG into a graph embedding, it is first necessary to obtain the corresponding instruction vector by looking up the *x2v* dictionary (“Common vector space embedding”). Then utilizes the LSTM with a self-attention mechanism to complete the basic block embedding (“Basic block embedding”), and its embedding result is used as the initialized feature vector of the graph node, finally Structure2Vec is used to generate a graph embedding $\vec {F^g}$ based on basic block embedding and adjacency matrix (“Graph embedding”).

#### Naming function embedding

Based on a pre-trained *k*2*v* dictionary, an LSTM carrying a self-attentive mechanism is used, which converts a naming function sequence into a naming function embedding $\vec {F^k}$ (“Naming function embedding”). The LSTM is chosen for its ability to capture the long sequences of subtokens composing naming function, and the self-attentiveness mechanism is chosen both for its ability to capture the most important naming semantics. Our experiments have confirmed that these mechanisms can significantly improve performance.

The graph embedding $\vec {F^g}$ and the naming function embedding $\vec {F^k}$ are put together to represent the binary function embedding result $\vec {F_{final}}$ defined in (2)

2 $$\begin{aligned} {\vec {F_{final}} = {concat(\vec {F^g},\vec {F^k})} } \end{aligned}$$

### Evaluation criteria

Once a function *F* is represented by an embedding $\vec {F}$ , the similarity between two binary functions can be converted into a comparison between vectors. We say $F_1\simeq F_2$ is similar if and only if $\vec {F_1}\simeq \vec {F_2}$ is similar. Thus, the key to binary code similarity is to find a suitable model $\Psi$ to transform a binary function into a vector. Given the binary functions $F_1^{s1}$,$F_2^{s1}$and $F_3^{s2}$, cosine is used as the similarity measure such that $cosine(\Psi (F_1^{s1}),\Psi (F_2^{s1}))\simeq 1$, and $cosine(\Psi (F_1^{s1}),\Psi (F_3^{s2})) \simeq -1$.

To generate the training dataset needed for model $\Psi$, we define two binary functions compiled from the same source code as similar and set *label* = +1 indicates they are similar, otherwise set *label* = − 1. During each round of training, the model parameters are adjusted and the $\Psi$ is updated continuously so that distance is minimized defined in (3), where $\varepsilon = cosine(\Psi (F_1),\Psi (F_2))$. The smaller the distance, the more accurate the $\Psi$ prediction, and vice versa. Similar to SAFE and UFEBS, accuracy was used as a model evaluation metric

3 $$\begin{aligned} {distance = \tfrac{1}{n}\sum _{i=1}^n (label- \varepsilon )^2 } \end{aligned}$$

To assess the quality of reproduction of the model $\Psi$ transformation, the *hits@N* evaluation metric is introduced as search precision of binary functions cross-platform and cross-optimization levels. In the binary code search scenario, the search results are a ranked list of the functions with the highest similarity. Assuming that the total number of binary functions from the same source code is *RightTotal*. For *N* results returned by a *query*, if there is a *result* and *query* that are both compiled from the same source code, add 1 to *RightCount*. The result of comparing *RightCount* and *RightTotal* is *hits@N* defined in (4)

4 $$\begin{aligned} {hits@N = \tfrac{RightCount}{RightTotal} } \end{aligned}$$

Take the vulnerability search as an example, suppose the source code carrying the vulnerability is compiled to generate 12 binary functions, using *A2* as a *query*, after manual verification in the returned results top50, the *RightCount* value is 8, so the hits@50 = 66.67%. Therefore, for the same *N*, higher *hits@N* means better search results and search results obtained by artificial intelligence technology are more reliable.

The *mAP* (mean Average Precision) defined in (5) is introduced to evaluate the model’s average prediction results for binary functions

5 $$\begin{aligned} {{mAP} = \tfrac{\sum _{q=1}^Q H_n(q)}{Q} } \end{aligned}$$

where *q* is the *q-th* prediction number, *Q* is the total number of predictions, and $H_n(q)$ is the *hits@N* precision of the *q-th* prediction result.

## N_Match implement

This section focuses on N_Match model implementations, respectively the common vector space embedding for cross-platform instructions (“Common vector space embedding”), basic block embedding (“Basic block embedding”), graph embedding (“Graph embedding”), naming function embedding (“Naming function embedding”) and Siamese network model (“Siamese network”*).

### Common vector space embedding

Different platform binary functions compiled from the same source code show that low-level assembly instruction syntax features heterogeneous and high-level semantic correlation, in other words, they are completely different in syntax, but express the same semantics. To solve the similarity comparison problem, we pre-trained a *x2v* language model that maps different platform instructions to the common vector space. After lifting to the common vector space, the meanings represented by each instruction dimension are the same, so the embedding results obtained by the deep learning model are comparable and trustworthy.

Instructions consist of opcodes and operands. Due to the diversity of operands such as offset addresses and immediate numbers, not only does OOV (Out Of Vocabulary) occurs in the pre-trained language model but also degrades the quality of embedding if they are not normalized. Thus we do not consider the original instruction as a word, but a normalized representation of the instruction as a word using Algorithm 1.
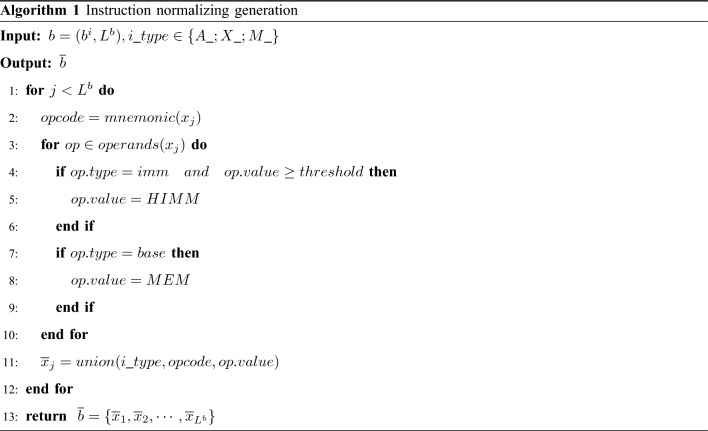


Specifically, similar to^5^, the platform type $i\_type\in \{A\_; X\_; M\_\}$ is first identified. Secondly, *base* address of the instruction is replaced by the special symbol *MEM*, e.g. an X86 instruction *mov eax,[0x3456789]* is replaced by *X_mov_eax,_MEM*. Immediate numbers over a certain range are replaced by the symbol *HIMM*, e.g. an ARM instruction *b 0x4018c0* is replaced by *A_b_HIMM*. Immediate numbers in a certain range remain unchanged, e.g. a MIPS instruction *daddiu t0, zero, 1* is replaced by *M_daddiu_t0,_zero,_1*. Finally, one unknown instruction is introduced for each instruction set, namely *A_UNK*, *X_UNK*, and *M_UNK*.

The input of Algorithm 1 is a basic block *b*, whose length is $L^b$ and the platform type of instruction is *i_type*. Extract the opcode of each instruction (line 2), then traverse all the operands of instruction (line 3) and determine the operand type, if the operand type is immediate and above threshold (lines 4–6) replace it with the symbol *HIMM*. If the operand type is a *base* address then the symbol is replaced by *MEM*(lines 7–9). Finally, the instruction platform type, opcode, and operand are concatenated together and returned (lines 11–13).

### Basic block embedding

Before completing the binary function graph embedding, the basic block nodes in the graph need to transform the feature vectors, and this work exists in the Gemini and UFEBS schemes as well. Since LSTM is more adapted to sequential data and has been proved in^16^ to have excellent results in basic block embedding, and also the attention mechanism can capture the important instructions of the basic block, we use LSTM carrying the attention mechanism to complete the basic block embedding. The basic block embedding results are used as feature vectors of nodes in the graph to provide node initialization for graph embedding. Similar implementations are included in previous works^17,18^. The basic block embedding is specified in Algorithm 2.
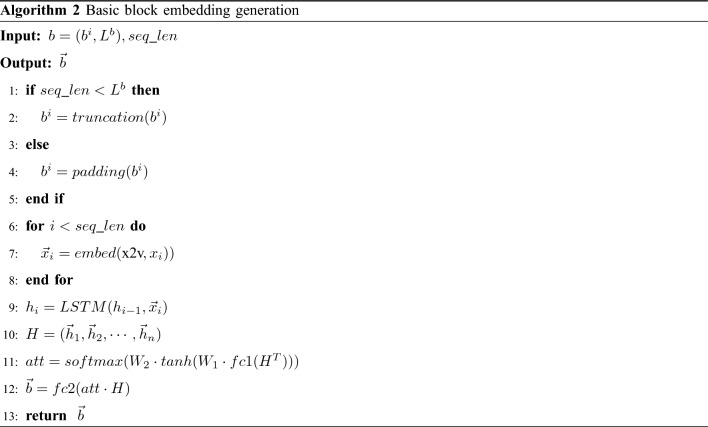


Lines 1–5, the basic blocks whose length exceeds *seq_len* are truncated, otherwise are padded. Next, each instruction embedding is looked up from the same space semantic *x2v* dictionary to generate a sequence of instruction embedding $(\vec x_1,\vec x_2,\ldots ,\vec x_n)$ (lines 6–8), then fed into the LSTM to obtain the hidden state $H = (\vec h_1,\vec h_2,\ldots ,\vec h_n)$ of instruction (lines 9–10), where *H* is a $seq\_len\times n$ matrix, *n* is the vector size of the hidden layer.

Subsequently, the self-attention weight matrix *att* is calculated (line 11). A fully connected layer *fc1* is operated on after the transpose to achieve a linear transformation from one feature space to another, which can improve the classification effect, and then the attention is calculated using a two-layer neural network based on each instruction position. where $W_1$ is a $h \times h$ matrix, *h* is the dense size, and $W_2$ is another ${h\times 1}$ matrix.

Finally, the weight matrix *att* is multiplied with *H* to obtain the basic block embedding and mapped to another feature space via a fully connected network *fc2* (line 12), providing an initialize node feature representation for the graph embedding.

### Graph embedding

Similar to the Gemini and UFEBS, based on the Structure2vec graph model inference algorithm, we update node representations with the aggregation results of neighborhood features. After *K* rounds of propagation, each graph node will generate a new real value vector that takes into account both graph characteristics and long-range interactions between nodes. Finally, we aggregate the embedding results of all graph nodes as the graph embedding. The CFG graph embedding is specified in Algorithm 3.
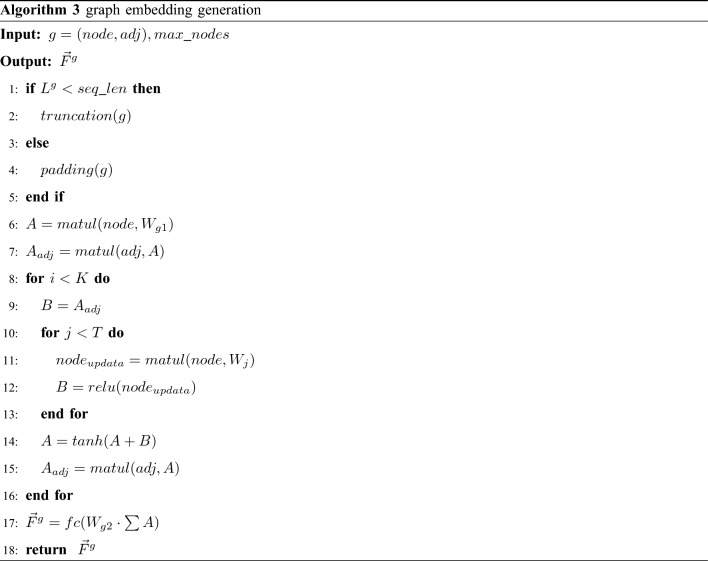


Make the number of basic blocks in the CFG equal to *max_nodes* by truncating or padding (lines 1–5). Lines 6–7 indicate initialization of nodes and message exchange, where *node* is a $max\_nodes \times h$ matrix and $W_{g1}$ is a $h \times o$ weight matrix, *h* is dense size and *o* is graph size, *adj* is a $max\_nodes \times max\_nodes$ matrix. The multiplication of *adj* and *A* is equivalent to a message exchange to achieve aggregation of neighboring nodes and represent it as $A_{adj}$, however, $A_{adj}$ does not contain the nodes themselves and does not implement node updates.

Lines 10–12 using the *leaky_relu* function to activate and map it to another semantic space by adding a *T* layer neural network. A variant of the *Relu* activation function, *leaky_relu*, is introduced here because the neuron cell cannot update the parameters when the *Relu* input is negative. In our implementation, *T=2* is set.

Lines 14–15 update the nodes and message exchange. Neighboring feature *B* and node feature *A* are updated by an additive operation to generate a new node representation and a new message exchange matrix which is subsequently assigned to *B* for the next iteration (line 9).

After *K* iterative operations, the basic block embedding is aggregated to obtain the graph embedding $\vec {F}^g$(line 17), which is mapped to another feature space via a fully connected network to facilitate the code similarity calculation.

### Naming function embedding

Inspired by^11^, we sequentially identify, extract and slice the API name carried on the binary code in linear address order to form a sequence of subtoken, then fed into an LSTM neural network with a self-attention mechanism to generate a naming function embedding. A binary naming function embedding is described in Algorithm 4.
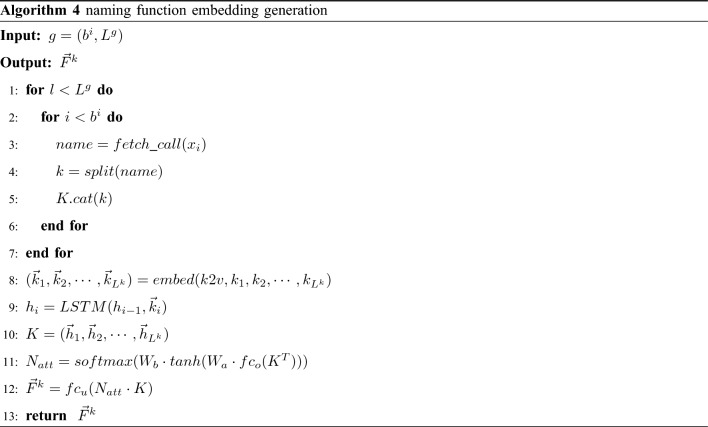


Lines 1–7 traverse all the basic blocks on the CFG, extracting the API or called function name carried by the call instruction (line 3) and representing the result as *name*. If the call instruction does not carry any naming information, it is indicated by the special symbol *noapi*, ensuring that any binary function carries a *name*. The longer the naming function the more knowledge it carries. However, if a long name is treated as one word, OOV problems may occur during pre-training. So longer names are sliced (line 4) to produce the subtoken sequence (line 5).

For experienced software engineers, APIs or defined function names usually have certain rules such as *camelcase* or *snake*. Since the rules are relatively clear, we do not adopt the BPE (Byte Pair Encoding)^19^ used in the^20^, but analyze these rules to slice the longer name into multiple subtokens. Specifically, names satisfying the *camelcase* rule are sliced according to capital letters, e.g. *flagWithResult* is sliced into *flag*, *with* and *result*, and the *snake* rule are sliced according to underscores, e.g. *Set_Flag_With_Result* is sliced into *set, flag, with* and *result*. Finally, the subtoken sequence is fed into LSTM carrying the self-attention mechanism to generate naming function embedding (lines 8–12).

### Siamese network

**Figure 4:**
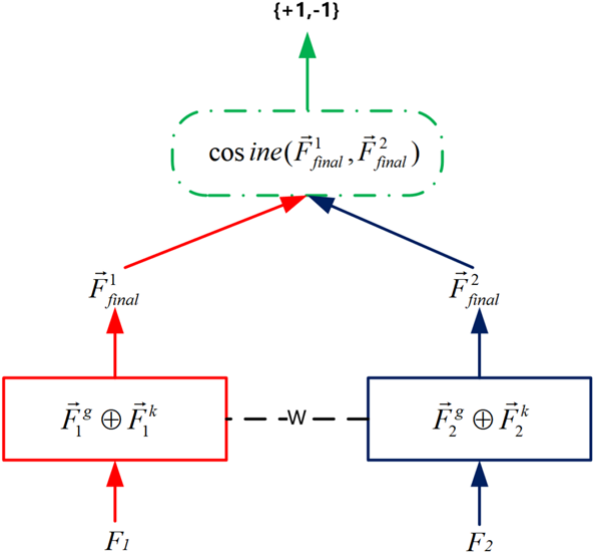
Architecture of Siamese network.

Siamese Network^21^ are widely used in binary code similarity computation^4,5,16^. We concatenate graph embedding and naming function embedding together as final binary function embedding, formally represented as $\vec {F}_{final} = \vec {F}^g \bigoplus \vec {F}^k$. Then, Siamese Network architecture sharing the same network parameters and weights is placed on top of two binary functions embedding (Fig. 4).

First, for a given pair of functions $F^1$ and $F^1$ to be compared, the function embedding $\vec {F}_{final}^1$ and $\vec {F}_{final}^2$ are obtained using N_Match. Then similarity between function embedding is calculated and defined in (6). The $W_{g1},W_a, W_b$ and $W_j$ model parameters are continuously optimized by the stochastic gradient descent algorithm to minimize objective functions defined in (7)

6 $$\begin{aligned} {sim(F^1,F^2)=cosine(\vec {F}_{final}^1,\vec {F}_{final}^2) }\end{aligned}$$

7 $$\begin{aligned} { min\sum _{i=1}^M (label - sim(F^1,F^2)) } \end{aligned}$$

## Experiment and evaluation

This section evaluates and analyses the results of N_Match against the baseline model in terms of accuracy, cross-platform, cross-optimization, vulnerability detection, and real scenario search, and some interesting observations are given based on the study of binary code similarity representation learning.

### Details

We implement N_Match with PyTorch which consists of data preprocessing, language pre-training models, and deep learning models.

#### Data preprocessing

We get the dataset in two ways. One is to download open-source software and compile it using a compiler with the -g option. The second is to use a publicly available binary dataset. Dataset preprocessing is performed using Radare2 to extract instructions and naming functions respectively. Pre-processing of the data showed large differences between the binary functions in terms of the number of basic blocks, the number of instructions, and the number of subtokens. Taking the OpenSSL dataset statistics as an example, excluding functions introduced by the compilation process in 610 binaries based on DWARF, it was found that on average each binary file contains about 8 functions, each function contains 8 basic blocks, with a maximum of 289 basic blocks, and basic blocks consist of 6 instructions, with a maximum of 5229 instructions. In terms of naming function, get an average of 29 subtokens per function, with a maximum of 807 subtokens.

#### Pre-training model

The language pre-training model uses the Word2Vec technique in gensim, including the *x2v* instruction pre-training model and the *k2v* naming function pre-training model.

1. ***x2v*** Since the Skip-gram strategy outperforms the CBOW strategy in terms of accuracy and low-frequency words, we generate instruction embedding vectors based on Skip-gram’s Word2vec model for datasets from three platforms (ARM, X86, MIPS), trained with the same configuration parameters. We make use of the dataset provided in the^22^ and an *i2v* dictionary^16^ containing ARM and X86 instructions provided in the^5^. The 32-bit and 64-bit binaries on different platforms with different compilers and different optimization levels were compiled and generated, using the Radare2 tool to form the three datasets. The parameters of the model are as follows: frequency is 8, window size is 8, and word embedding size is 100. Lastly, the dictionaries from the three models are integrated to obtain a final *x2v* dictionary with a capacity of 696,779 $\times$ 100.
2. ***k2v*** Similar to *x2v*, Word2vec was used to generate a *k2v* dictionary. The dataset uses 464 binary files provided in the^11^, and function simple path extraction is performed using Algorithm 4 to generate a dataset containing 84 million lines fed into the Word2vec model for training. The parameters of the model are as follows: subtoken embedding size is 96, the window size is 5, and frequency is 5. Finally, a *k2v* dictionary containing 8756 $\times$ 96 subtokens is obtained. The original naming function is seen as set *A*, and the subtoken is seen as set *B*. Statistical analysis of the dataset shows that the ratio of the number of elements in set *A* to set *B* is 36.3%. The reason for the decrease in proportion is that the *snake* and *camelcase* semantics are made up of subtoken that can reduce the dictionary size.

#### Deep learning model

N_Match deep learning model using PyTorch framework. Same to UFEBS^5^, the instruction length within the basic block $L^b = 50$, the number of basic blocks $L^g = 150$, the learning rate is 0.001, the optimizer is *Adam*, the loss function is *MSE* (Mean Square Error). The graph embedding and function named embedding size are 64, choosing the embedding size to be 64 is a good trade-off between the performance and efficiency^4,5^, so the final function embedding size is 128. In the Structure2vec model, the iterative number of rounds $K = 2$, a number of layers $T = 2$, and a single layer LSTM cell is used for the basic block embedding and naming function embedding.

Our experiments are conducted on the ubuntu16 operating system with two Intel Xeon E5-2650v4 CPUs (28 cores in total ) running at 2.20 GHz, 128 GB memory, and a Tesla P100 GPU card. During both training and evaluation, only 1 GPU card was used.

### Baseline

Several previous works have been proposed for binary code similarity cross-platform search problems, such as^17,23^. Since Gemini^4^ and UFEBS^5^ achieve cross-platform similarity with the help of Structure2vec graph neural networks, which outperform other solutions in terms of accuracy and efficiency. Therefore, Gemini and UFEBS are used as baselines for model evaluation and comparison with N_Match. Our N_Match scheme is also compared with SAFE^16^ due to the inherently linear address sequence of binary, which it is more convenient to use RNN for function embedding.

Gemini^4^ uses a manual approach to extract basic block features which is a supervised feature extraction scheme. Each basic block is represented as a numeric feature vector. All the basic block features and an adjacency matrix of CFG are then fed into the Structure2vec to obtain the graph embedding. As the basic block feature is not modified by the training procedure of the neural network, it cannot be trained.

UFEBS^5^ uses Word2Vec to train instruction pre-training models for both X86 and ARM platforms respectively which is an unsupervised feature extraction scheme. GRU units carrying a self-attention mechanism are used to generate the basic block embedding. Subsequently, the basic block embedding and the adjacency matrix of CFG are fed into the Structure2vec to obtain the graph embedding.

SAFE^16^ uses the pre-trained *i2v* model and bidirectional GRU to generate a function embedding, then fed into a Siamese network with shared weights for model training. SAFE is also an unsupervised feature extraction scheme.

As UFEBS and SAFE utilize *i2v* to achieve similar computation between ARM and X86 only, we extend *i2v* to become *x2v* as a pre-trained model so that it supports MIPS, ARM, and X86.

### DataSets

For descriptive convenience, the symbol *Pn* is introduced to denote a binary function at a certain platform with optimization level, where $P \in \{X, A, M\}$ represents the platform environment on which the binary function runs, and *n* represents the optimization level, e.g. *X3* represents the binary function generated using optimization level -O3 for X86 platform.

#### Model train datasets

Similar to the dataset used by Gemini and UFEBS, DataSet_A and DataSet_B are used for binary code similarity model training. Both DataSet_A and DataSet_B are compiled using the GCC compiler to generate binaries for different platforms, carrying the -O[0,1,2,3] optimization level. The training set, validation set, and test set are generated in the ratio of 80%:10%:10%.

DataSet_A: Compile the source code *OpenSSL1.1* for two versions (*k* and *m*) to generate binary functions for X86, ARM, and MIPS platforms, resulting in a total of 730,458 pairs of functions.

DataSet_B: We compiled the open source code *OpenSSL1.1.f* to generate both X86 and ARM platforms. Here, we collect only functions with more than 5 basic blocks. In total, DataSet_B contains 126,786 pairs of functions.

#### Model evaluation datasets

DataSet_I, DataSet_II, DataSet_III, and DataSet_IV evaluate model accuracy at a granular level for cross-platforms and cross-optimization levels. Datasets are obtained from the^22^, including not only some files used by the training dataset, but also single file, individual software, and entire *coreutils* kernels program. The datasets are all generated binaries using the GCC8.2 compiler and carrying -O[0, 1, 2, 3] optimization levels for 64-bit X86, ARM, and MIPS platforms.

DataSet_I: This dataset is compiled for only one *dir* component of *coreutils6.7*, resulting in a total of 12 binaries.

DataSet_II: This dataset compiles the entire open source software *coreutils6.7* consisting of 94 components such as *dir* and *ls*, resulting in a total of 1128 binaries.

DataSet_III: The dataset compiled by the open source software *busybox1.21*, resulting in a total of 12 binaries.

DataSet_IV: The dataset compiled by the open source software *libssl*, resulting in a total of 12 binaries.

#### Function search database

DataSet_V is a function search database for evaluating model search performance. The database is composed of *which, time, tar, inetutils, gzip, findutils, coreutils* etc. Compiled with CLANG (7.0/6.0/5.0/4.0) and GCC (8.2/7.3/6.4/5.5/4.9.4) compilers, carrying industry widely used -O[2,3] optimization level, to generate 2916 binary files on 64-bit ARM, MIPS, X86 platforms, then extracting 1,002,913 binary functions with the help of Radare2.

#### Vulnerability database

A source code with vulnerability compiled to generate 12 binary functions, which is used to evaluate the *mAP*.

### Model accuracy

This experiment uses DataSet_A and DataSet_B to evaluate the accuracy of N_Match compared to the baseline model. We set epochs = 10, and shuffle the training dataset in each epoch. At the end of each epoch, the validation accuracy $A_{val}$ is calculated on the validation dataset. if the current $A_{val}$ is the highest, the test dataset calculation is performed to obtain the test accuracy $A_{test}$. In this way, the best accuracy $A_{val}$ and $A_{test}$ are obtained for each model. The results of model training are shown in Table 2.

**Table 2:** Model accuracy.

Model	$A_{val}$	$A_{test}$	DataSet
SAFE	0.8844	0.8855	DataSet_A
UFEBS	0.7956	0.7937	DataSet_A
N_Match	0.9226	0.9228	DataSet_A
Gemini	0.6986	0.6914	DataSet_B
SAFE	0.9155	0.9140	DataSet_B
UFEBS	0.7822	0.7802	DataSet_B
N_Match	0.9791	0.9793	DataSet_B

As found in Table 2, N_Match outperformed the SAFE and UFEBS by achieving $A_{val}$ of 92.26% and $A_{test}$ of 92.28% on the DataSet_A. On the DataSet_B, N_Match also performed well with a validation accuracy of 97.91% and a test accuracy of 97.93%. N_Match model is 19% more accurate than UFEBS and 30% more accurate than the Gemini. It is shown that the fusion of naming function embedding and graph embedding can improve model accuracy, indicating that stable and platform-independent features can greatly mitigate cross-platform variation.

It is found that Gemini performs the worst, with an accuracy rate of 69%. The reason is that the Gemini scheme^4^ is more adapted to function pairs with more than 20 basic blocks. On the DataSet_A, the SAFE accuracy is 9% higher than the UFEBS scheme. The reason may be that RNNs are good at learning representations of time-series instructions, while graph neural networks need to be improved in handling CFG composed of all possible paths.

In the following binary code analysis task, we will conduct experimental analysis around the model trained on the DataSet_A.

**Table 3:** Accuracy of cross-platform.

Task	N_Match	SAFE	UFEBS	N_Match	SAFE	UFEBS
	DataSet_I			DataSet_II		
X0–A0	0.9367	0.5292	0.8425	0.9338	0.5305	0.8221
A0–M0	0.8767	0.4768	0.8901	0.8788	0.4470	0.8692
X0–M0	0.8996	0.8404	0.8834	0.9040	0.8450	0.8684
	DataSet_III			DataSet_IV		
X0–A0	0.9687	0.5841	0.9284	0.8823	0.8793	0.8119
X0–M0	0.8828	0.7494	0.9272	0.9770	0.8508	0.8543
M0–A0	0.8567	0.5044	0.9301	0.9043	0.9071	0.8425

### Cross-platform comparison between the same optimization levels

The task is to evaluate the accuracy of the same source code for binary functions on different platforms with the same compiler and same optimization level. Specifically, we extract A0, M0, and X0 function pairs to evaluate the model accuracy of N_Match, SAFE, and UFEBS in a real environment, and 3 groups of comparison results are obtained from evaluation datasets. The results of the comparisons are shown in Table 3.

**Figure 5:**
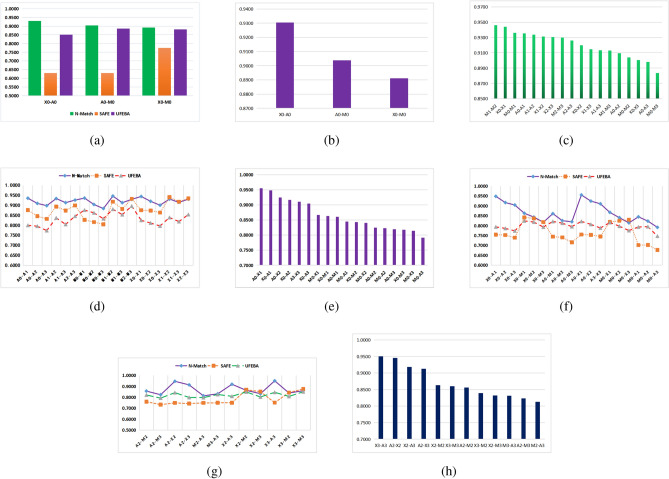
(**a**) Cross-platform compare accuracy between N_Match, SAFE and UFEBS using O0 as an example; (**b**) cross-platform compare accuracy of N_Match using O0; (**c**) cross-optimization compare accuracy of N_Match; (**d**) cross-optimization compare accuracy between N_Match, SAFE and UFEBS; (**e**) comparison between optimized and unoptimized of N_Match; (**f**) optimized and unoptimized compare accuracy between N_Match, SAFE and UFEBS; (**g**) O2 vs O3 comparison accuracy between N_Match, SAFE and UFEBS in industrial scenarios; (**h**) O2 vs O3 comparison of N_Match.

The comparison in Table 3 shows that N_Match outperformed SAFE and UFEB, with an average accuracy of 90.85% for N_Match, 67.87% for SAFE and 87.25% for UFEBS. Although SAFE accuracy is 9% higher than UFEBS accuracy on the DataSet_A (Table 2), the average UFEBS accuracy is 20% higher than SAFE accuracy on the real evaluation dataset (Fig. 5a), show that stable nature of the graph structure improves the similarity results. We calculate the average of the accuracy results of the N_Match in four data sets. The results show that X86 and ARM are the most similar, and the accuracy of X0–A0 is 4% higher than that of X0–M0 (Fig. 5b).

### Comparison of the same platform across optimization levels

This task is to evaluate the accuracy of functions with different optimization levels for the same compiler and the same platform using DataSet_III and DataSet_IV. Since each platform can generate 4 functions at different levels of optimization, each platform is 6 groups of comparison results for a total of 18 groups, and the results of the comparison are shown in Table 4.

**Table 4:** Accuracy of cross-optimization levels.

Task	DataSet_III			DataSet_IV		
	N_Match	SAFE	UFEBS	N_Match	SAFE	UFEBS
A0–A1	0.9503	0.8518	0.8425	0.9204	0.8998	0.7570
A0–A2	0.9115	0.8000	0.8349	0.9074	0.8914	0.7561
A0–A3	0.8864	0.7786	0.8045	0.9099	0.8854	0.7426
A1-A2	0.9426	0.8329	0.8727	0.9246	0.9543	0.8009
A1-A3	0.9042	0.7996	0.8358	0.9220	0.9479	0.7734
A2-A3	0.9150	0.8418	0.8614	0.9369	0.9567	0.8299
M0–M1	0.9144	0.7506	0.8687	0.9575	0.9035	0.8831
M0–M2	0.8594	0.7237	0.8402	0.9484	0.9083	0.8843
M0–M3	0.8213	0.7109	0.8053	0.9462	0.8992	0.8602
M1-M2	0.9129	0.8798	0.8863	0.9794	0.9555	0.8746
M1-M3	0.8636	0.8156	0.8381	0.9621	0.9479	0.8682
M2-M3	0.8778	0.8841	0.8667	0.9817	0.9805	0.9253
X0–X1	0.9539	0.8293	0.8542	0.9342	0.9216	0.7959
X0–X2	0.9178	0.8307	0.8440	0.9220	0.9167	0.7788
X0–X3	0.8877	0.8134	0.8132	0.9130	0.9142	0.7800
X1-X2	0.9434	0.9376	0.8747	0.9188	0.9435	0.8032
X1-X3	0.9093	0.8956	0.8434	0.9200	0.9395	0.7937
X2-X3	0.9330	0.9247	0.8765	0.9284	0.9443	0.8310

The comparison in Fig. 5d shows that N_Match performed well on all 18 groups of comparison results with an average accuracy of N-Match 92.05%, that of SAFE is 87.81%, and that of UFEBS is 83.34%. The closely related optimization levels are more similar to each other, with O1 and O0 having the highest accuracy, followed by O1 and O2, and O0 and O3 being the least similar, as shown in Fig. 5c. This is because O1 is a basic optimization based on O0 and O2 is an upgraded version based on O1, thus presenting the above results.

In Table 4, it is found that the accuracy of SAFE is higher than that of N_Match in a few optimization levels, and also found that the accuracy of SAFE outperforms UFEBS in most optimization levels as shown in Fig. 5d, indicating that the sequence model outperforms the graph model for some optimization levels such as P2–P3. Overall, N_Match has the best model accuracy in comparisons of cross-optimization levels with the same platform.

### Optimized and unoptimized

This task evaluates the accuracy of the model between unoptimized -O0 functions and optimized functions using DataSet_III and DataSet_IV. Each platform is 6 groups of results for a total of 18 groups, and the comparison results are shown in Table 5.

**Table 5:** Accuracy of cross-optimization levels.

Task	DataSet_III			DataSet_IV		
	N_Match	SAFE	UFEBS	N_Match	SAFE	UFEBS
X0–A1	0.9480	0.5989	0.8416	0.9489	0.9098	0.7460
X0–A2	0.9035	0.6118	0.8260	0.9299	0.8936	0.7465
X0–A3	0.8850	0.5823	0.8134	0.9239	0.8955	0.7337
X0–M1	0.8392	0.7611	0.8493	0.8863	0.9219	0.7988
X0–M2	0.7901	0.7614	0.8504	0.8964	0.9125	0.7862
X0–M3	0.7347	0.7201	0.7868	0.9000	0.9161	0.7989
A0–M1	0.8169	0.5715	0.8522	0.9049	0.9175	0.7903
A0–M2	0.7773	0.5816	0.8479	0.8727	0.8989	0.7773
A0–M3	0.7589	0.5300	0.7990	0.8805	0.9019	0.7912
A0–X1	0.9464	0.5937	0.8438	0.9643	0.9166	0.7995
A0–X2	0.9012	0.5981	0.8334	0.9472	0.9084	0.7797
A3-X3	0.8791	0.5918	0.8129	0.9421	0.8987	0.7633
M0–X1	0.8415	0.7632	0.8411	0.8924	0.8741	0.7907
M0–X2	0.8068	0.7570	0.8211	0.8741	0.8927	0.7737
M0–X3	0.7657	0.7544	0.7791	0.8624	0.9052	0.7713
M0–A1	0.8220	0.5177	0.8362	0.8674	0.8861	0.7504
M0–A2	0.7985	0.5097	0.8301	0.8471	0.8949	0.7600
M0–A3	0.7462	0.4886	0.7774	0.8367	0.8659	0.7167

The comparison results show that the average accuracy of N_Match is 86.5%, SAFE is 76.4% and UFEBS is 79.77%. Except for the group of M0–X3, N_Match performs well on the remaining 17 groups of comparison results, as shown in Fig. 5f. Comparing the N_Match model for the same platform (Fig. 5c) and different platforms (Fig. 5e) shows that the similarity results of the optimization levels vary significantly cross-platforms. The top 5 ranking in Fig. 5e confirms that ARM and X86 are the most similar, and O1 and O0 have the highest similarity.

As seen in Fig. 5f, SAFE accuracy is lower than UFEBS accuracy at multiple optimization levels, indicating that graph structure stability helps to improve similar accuracy of cross-platforms and cross-optimization. The more stable the knowledge, the more similar the binary functions corresponding to the same source code on cross-optimization and cross-platform.

It is also found in Table 5 that N_Match is inferior to SAFE and UFEBS in comparing X0 with MIPS platforms, A0 with MIPS platforms, M0 with ARM platforms, and M0 with X86 platforms. Analyzing the reasons for this phenomenon, it is possible that the N_Match system does not fully recognize the MIPS call instructions and thus does not capture the semantics of the system calls contained in the functions.

### Industrial scenarios

This task evaluates the accuracy of O2 and O3 comparisons in industrial scenarios using DataSet_III and DataSet_IV as datasets, for a total of 12 groups of comparison results, as shown in Table 6.

**Table 6:** Accuracy of cross-optimization levels.

Task	DataSet_III			DataSet_IV		
	N_Match	SAFE	UFEBS	N_Match	SAFE	UFEBS
A2-M2	0.8369	0.6082	0.8930	0.8758	0.9104	0.7482
A2-M3	0.7825	0.5451	0.8235	0.8636	0.9199	0.7608
A2-X2	0.9553	0.6157	0.8923	0.9364	0.8812	0.7919
A2-X3	0.9090	0.6100	0.8364	0.9169	0.8743	0.7595
M2-A3	0.7771	0.5867	0.8330	0.8491	0.9097	0.7607
M3-A3	0.8236	0.5800	0.8956	0.8384	0.9199	0.7547
X2-A3	0.9144	0.6110	0.8508	0.9230	0.8876	0.7668
X2-M2	0.8458	0.8150	0.8911	0.8810	0.9220	0.8061
X2-M3	0.7949	0.7859	0.8318	0.8699	0.9161	0.7734
X3-A3	0.9645	0.6016	0.9073	0.9361	0.8995	0.7827
X3-M2	0.7863	0.7603	0.8313	0.8915	0.9260	0.7837
X3-M3	0.8295	0.8126	0.8908	0.8899	0.9381	0.8093

**Table 7:** Model accuracy.

Task	h@200	h@100	h@50	h@20	h@15	h@12	*mAP*
M3	1.0000	0.9167	0.7500	0.7500	0.7500	0.7500	0.8194
M0	0.7500	0.5833	0.5000	0.4167	0.4167	0.4167	0.5139
A1	1.0000	1.0000	1.0000	0.9167	0.9167	0.8333	0.9444
X3	0.8333	0.8333	0.6667	0.5000	0.3333	0.2500	0.5694
X2	1.0000	0.9167	0.9167	0.7500	0.6667	0.6667	0.8194
A0	0.9167	0.9167	0.9167	0.8333	0.7500	0.7500	0.8472
X1	1.0000	1.0000	1.0000	0.9167	0.9167	0.8333	0.9444
A3	0.8333	0.8333	0.6667	0.5000	0.2500	0.1667	0.5417
M1	0.5833	0.5000	0.2500	0.1667	0.1667	0.1667	0.3056
M2	1.0000	1.0000	0.8333	0.7500	0.7500	0.5000	0.8056
A2	1.0000	1.0000	0.9167	0.7500	0.7500	0.6667	0.8472
X0	0.9167	0.9167	0.9167	0.7500	0.7500	0.6667	0.8194
*mAP*	0.9028	0.8681	0.7778	0.6667	0.6181	0.5556	0.7315

The comparisons show that the average accuracy of N_Match is 87.05%, SAFE is 78.49% and UFEBS is 81.98%. No solutions are able to outperform all 66 compared solutions across three platforms. As shown in Fig. 5g, on the X2–M2, X2–M3, X3–M2, and X3–M3 comparison results, N_Match accuracy is not as good as SAFE, which shows that the learning scheme using instruction sequence representation has an advantage in the similarity comparison of industrial scenarios. From Fig. 5h, it is clear that the N_Match model has the highest comparison accuracy in comparing the same optimization level for ARM and X86 platforms, such as X3–A3 and X2–A2. Indicating that optimization options carried by query have a greater impact on computational results. Therefore, identifying the optimization of a binary function can be used as a filtering condition for the binary code similarity task.

### Vulnerability search

This task evaluates the *hit@N* of a query search using DataSet_III as the search database. Specifically, using a *Pn* as the query to return topN predictions from DataSet_III, which contains 33,286 binary functions.

We used CVE-2021-42374 as a *query*, which is located in the *unpack_lzma_stream*() of busybox software and contains 73 basic blocks (in the A2 binary function). As can be seen from the above that the same source code function generates 12 binary functions and therefore 12 known vulnerabilities in the Target database. For a query, *RightTotal* = 12 and *RightCount* is the true correct verified result manually in top N. Finally, *hit@N* and *mAP* are calculated based on (18) and (19).

Since SAFE and UFEBS have lower hit@N at N = 50, we only set N = 200, N = 100, and N = 50 for the experimental task and compared them, the results as shown in Table 8. Based on hit@N results, we compute the *mAP* metrics of the three models. The results show that N_Match far outperformed SAFE and UFEBS in terms of average *mAP*, with N_Match is 84.72%, SAFE is 12.73% and UFEBS is 18.29%. Even though UFEBS introduces a stable graph structure for similarity evaluation, the *mAP* of N_Match exceeds UFEBS by up to 66%. The vulnerability experiments illustrate that in the challenging task of binary code similarity comparison of cross-platform and cross-optimization levels, the current evaluation is only based on the model training accuracy, which still needs to be optimized and improved in the real industrial scene, additional evaluation metrics such as *mAP* should be introduced to assess the models comparison.

**Table 8:** Hit@N on vulnerability search scenarios.

Task	SAFE				UFEBS				N_Match			
	h@200	h@100	h@50	*mAP*	h@200	h@100	h@50	*mAP*	h@200	h@100	h@50	*mAP*
M3	0.1667	0.1667	0.0833	0.1389	0.0833	0.0833	0.0833	0.0833	1.0000	0.9167	0.7500	0.8889
X3	0.1667	0.1667	0.1667	0.1667	0.1667	0.1667	0.0833	0.1389	0.8333	0.8333	0.6667	0.7778
M0	0.2500	0.1667	0.0833	0.1667	0.2500	0.2500	0.2500	0.2500	0.7500	0.5833	0.5000	0.6111
X2	0.0833	0.0833	0.0833	0.0833	0.2500	0.0833	0.0833	0.1389	1.0000	0.9167	0.9167	0.9445
X1	0.1667	0.1667	0.0833	0.1389	0.1667	0.1667	0.0833	0.1389	1.0000	1.0000	1.0000	1.0000
A1	0.0833	0.0833	0.0833	0.0833	0.2500	0.1667	0.1667	0.1945	1.0000	1.0000	1.0000	1.0000
A0	0.0833	0.0833	0.0833	0.0833	0.2500	0.2500	0.2500	0.2500	0.9167	0.9167	0.9167	0.9167
M2	0.1667	0.1667	0.1667	0.1667	0.5000	0.0833	0.0833	0.2222	1.0000	1.0000	0.8333	0.9444
M1	0.1667	0.1667	0.1667	0.1667	0.4167	0.0833	0.0833	0.1944	0.5833	0.5000	0.2500	0.4444
A2	0.0833	0.0833	0.0833	0.0833	0.2500	0.1667	0.0833	0.1667	1.0000	0.9167	0.9167	0.9445
X0	0.1667	0.1667	0.1667	0.1667	0.2500	0.2500	0.2500	0.2500	0.9167	0.9167	0.9167	0.9167
A3	0.0833	0.0833	0.0833	0.0833	0.1667	0.1667	0.1667	0.1667	0.8333	0.8333	0.6667	0.7778
*mAP*	0.1389	0.1320	0.1111	0.1273	0.2500	0.1597	0.1389	0.1829	0.9028	0.8611	0.7778	0.8472

We continue to evaluate the N_Match model by setting N = 200, 100, 50, 20, 15, and 12 respectively, calculating the hit@N of the 12 queries, and the results are shown in Table 7. Even in top12, N_Match has a *mAP* value of 55.56%, and is able to find more than half of the binary functions that are compiled from the same source code but different platforms and different optimization. Also found in top12, optimization level O1 was able to find more than 80% of vulnerabilities on ARM and X86.

**Figure 6:**
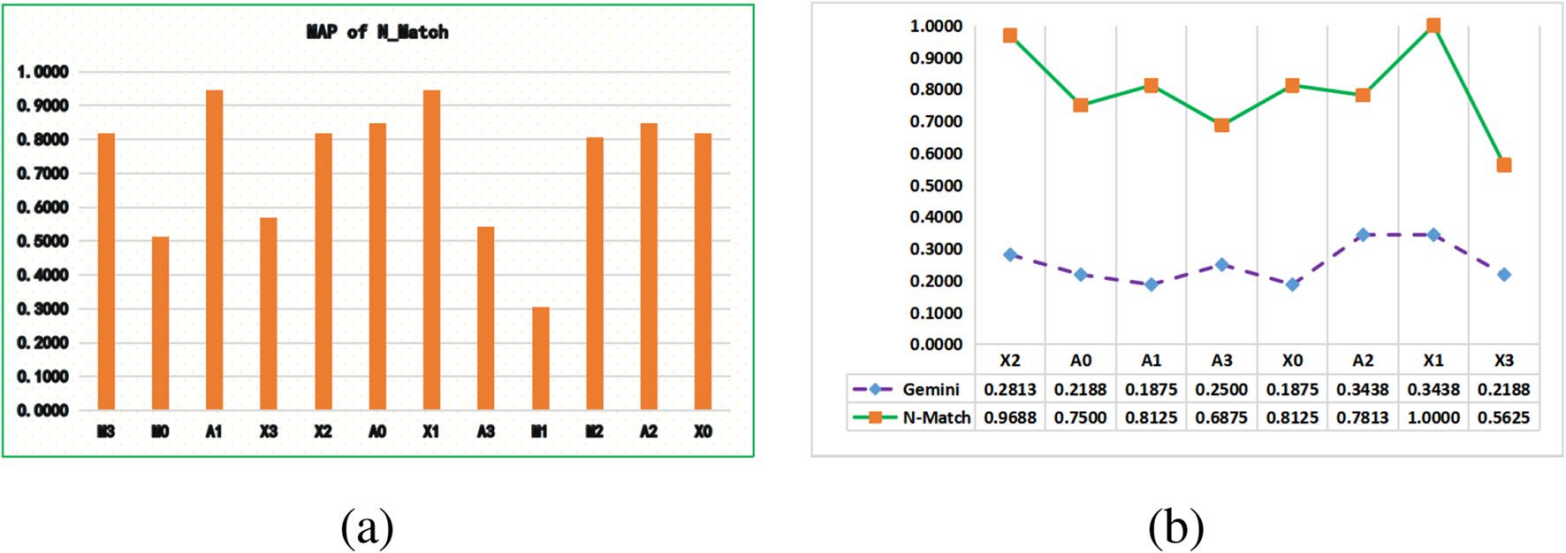
(**a**) *mAP* of N_Match; (**b**) *mAP* of N_Match and *mAP* of Gemini.

We also compared the *mAP* of 12 function searches (Fig. 6a). The statistical analysis found that A1 and X1 work best, and M0 and M1 work worst, with a 64% difference in *mAP*. On the same optimization level comparison, M2, X2, and A2 are smoother with a little gap of 80.56%, 81.94%, and 84.72% *mAP* respectively, however M3, X3, and A3 have a larger gap of 81.94%, 56.94% and 54.17% *mAP* respectively. This indicates that the O3 optimization level, which focuses on execution efficiency, may introduce more unpredictable program behavior such as errors and inline function, making the *mAP* unstable. Therefore O3 as *query* should be avoided when comparing similarities. We argue that reverse engineers should determine the optimization level and platform of *query* when searching for vulnerabilities. We also compare the search results of N_Match and Gemini (Fig. 6b). The N_Match outperforms the Gemini. Although Gemini and UFEBS also draw on graph structures, we argue that the graph structure may simply be stable semantics.

We performed a further comparison with the ComFU model proposed by us in^24^. ComFU does not utilize graph structure and employs data dependency analysis techniques to extract sequences of parameter slices around the APIs that trigger vulnerabilities within functions. The comparison reveals that ComFU filters out a large number of dissimilar functions, and the distance gap between real vulnerability functions and dissimilar functions is obvious. This suggests that vulnerability-oriented similarity detection, with functions as the comparison object of coarse granularity, is not as good as using the vulnerability key code fragments as the object.

### Function search performance

This task evaluates the model search performance on DataSet_V by comparing model training time $T_m$, function vector generation time $T_g$, and function search time $T_s$, which are used to guide the best industry practice of binary function search.

**Table 9:** Hit@N of N_Match.

Model	$T_m(m)$	$T_g(m)$	$T_s(s)$
N_Match	3590	313	227
SAFE	259	17	128
UFEBS	3900	347	128

As shown in Table 9, in terms of model training time, the graph model costs far more than the sequence model. The sequence model takes the shortest training time, which is 15$\times$ faster than the graph model. In terms of vector generation time, the sequence model is 20$\times$ faster than the graph model. However, the vector search time of graph model and sequence model is the same, the difference comes from the time of dimension of vector computation. Although the N_Match vector size is 128 which is twice that of UFEBS and SAFE, a function search time from 1 million vector library is negligible.

### Ablation study

In order to evaluate the contribution of the core components to our function representation, we compare the attention mechanism and graph neural network configurations.

In the evaluation of the attention mechanism, we compare GRU, RNN, LSTM, and Atttion+LSTM on sequences of instructions while the model was being trained, and the results are shown in Table 10. The comparison reveals that the accuracy of the model carrying the attention mechanism is 39% higher than that of the RNN model. This indicates that the attention mechanism can focus on the more critical information of the binary code similarity comparison task and improve the task accuracy. It is also found that Transformer is not as accurate as the LSTM that carries the attention mechanism. The reason for this may be analyzed because the Transformer lies in considering only attention, whereas instructions naturally have sequential meanings, or perhaps Transformer performs best when both encoding and decoding are employed simultaneously.

From the model comparison in Table 2, it is found that UFEBS with graph neural network is almost 9% lower than SAFE without graph neural network in terms of accuracy (see Table 2), but UFEBS is almost 6.5% higher than SAFE in terms of map metrics in terms of vulnerability matching (Table 8). This proves that graph neural network helps to reduce FPR.

**Table 10:** Components accuracy.

	GRU	RNN	Attion+LSTM	LSTM	Transformer
$A_{val}$	0.5917	0.5816	0.9689	0.5913	0.8820
$A_{test}$	0.5807	0.5746	0.9703	0.5822	0.6667

## Related work

This section presents work related to binary code similarity from the perspective of instruction sequence and CFG of binary function.

### Similarity based on instruction sequences model

The natural linear address layout of binary function provides advantages for sequence-based analysis schemes. The simplest way to do this is to hash a fixed-length sequence of instructions, and if the hashes are the same the sequences are considered similar. Qiao et al.^25^ infers function similarity by counting similar basic blocks. Jin et al.^26^ computes the input–output behavior within the basic block. Hashing techniques retrieve matches efficiently and are able to sense changes in code. Differences in instruction syntax or graph structure will cause huge changes.

Several works extend the basic blocks. David and Yahav^2^ extracts continuous basic blocks from the CFG to form a trace, and uses the instruction alignment method and the longest common string matching to calculate the similarity between traces. Huang et al.^3^ extracts the longest execution path on the CFG, establishes the basic block mapping relationship between two binary functions, and computes the similarity value. The above solutions are less accurate on small code fragments, and do not solve the cross-platform similarity calculation.

Based on Word2Vec^12^ or Doc2Vec^27^, recent works utilize recurrent neural networks to obtain a function sequence learning representation. Redmond et al.^17^ treats instruction as a word and uses RNN to achieve similarity comparison between basic blocks. Ding et al.^28^ treats operand and operand as word, only the X86 platform similarity calculation is achieved. Zuo et al.^18^ implements similarity comparison between basic blocks through the machine translation NMT (Neural Machine Translation) technique, which only implements similarity computation between X86 and ARM platforms.

### Similarity based on graph model

Current schemes based on CFG transform a CFG into a graph embedded with a graph neural network. Several works study code similarity based on CFG. Gao et al.^29^ constructs Labeled Semantic Flow Graphs and then extracts the feature vectors of each basic block to generate graph embedding. Yu et al.^30^ argues that the order of control flow graph nodes is important for graph similarity detection and using a BERT^31^ (Bidirectional Encoder Representations from Transformers) as a language per training model.

A number of schemes extend CFG. Alrabaee et al.^6^ combines CFG, function call graph, and register flow graph together into a semantic integrated graph. Zhang et al.^8^ argues that although the order of instructions has changed, the dependency graph between instructions remains the same and proposes a RIDG (Reductive Instruction Dependent Graph). Qiu et al.^7^ fuses inline functions and library functions called by CFG and proposes the EDG (Execution Dependence Graph).

## Conclusions and future works

In this paper, we propose a binary code similarity model N_Match that fuses naming function and graph neural network, which goal is to find as many binary functions as possible from the same source code at different optimization levels on different platforms. The core idea of the model is to leverage platform-independent naming function, common vector space, and stable graph structure semantics. We evaluate and compare the model on multiple datasets and show that N_Match outperforms current models in terms of accuracy, cross-platform, cross-optimization level, and hit@N. Real-world vulnerability search experiments verify that N_Match is able to match more binary functions with a higher *mAP*. We give some comparative conclusions between cross-platform and cross-optimization based on deep learning. To this end, we make our dataset, code, and trained models publicly available at https://www.github.com/CSecurityZhongYuan/Binary-Name_Match.

### Future works

In our future work, we can use the temporal and textual data of the nodes to compute the edge weights and then generate subgraphs with highly relevant nodes, and similarity comparisons are made through subgraphs^32^. Secondly, utilizing the abstract syntax tree of the pseudo-code which holds richer semantic information in each node, such as data types and statement execution order context information. Thirdly, variable names and their variable types can also express the semantics of the code. Based on big code^10^ and linguistic statistical models, with the help of neural network translation techniques, the recovered variable names and variable types^33^ will be used in code similarity. Finally, no matter how functionally identical code is changed, the core descriptions that determine the function of the code remain the same, and with the help of source code summarization^34^ generation techniques, a new means^11^ of comparing binary code naming descriptions is not lost.

## Data Availability

The data used to support the findings of the study are available at https://www.github.com/CSecurityZhongYuan/Binary-Name_Match.
